# Impact of Sex on Plasma Biomarkers in ob/ob Mice

**DOI:** 10.3390/ijms27041712

**Published:** 2026-02-10

**Authors:** Yunha Suh, Kwang-eun Kim

**Affiliations:** 1 Organelle Medicine Research Center, Yonsei University Wonju College of Medicine, Wonju 26426, Republic of Korea; 2 Department of Convergence Medicine, Yonsei University Wonju College of Medicine, Wonju 26426, Republic of Korea; 3 Department of Global Medical Science, Yonsei University Wonju College of Medicine, Wonju 26426, Republic of Korea; 4 Institute of Mitochondrial Medicine, Yonsei University Wonju College of Medicine, Wonju 26426, Republic of Korea

**Keywords:** sex differences, plasma proteome, biomarker, ob/ob, ENO2, NSE

## Abstract

Sex is a critical biological variable that influences disease incidence, progression, and therapeutic responses; therefore, it must be incorporated into biomedical research. Despite this, most mouse studies historically have not compared animals by sex. Recently, growing evidence has indicated that sex-specific analyses are important in obesity and metabolic disorders. The ob/ob mouse is a widely used model for metabolic disease research; however, sex differences in plasma biomarkers have not been fully characterized in this model. In this study, male and female ob/ob mice at 8 weeks of age exhibited comparable body weight, blood glucose levels, and adipose tissue mass. Plasma proteomics analysis using the Olink platform revealed that 27% (23/84) of quantified proteins exhibited sex differences, with 91% (21/23) of these proteins elevated in females. Notably, Enolase 2 (ENO2), also known as neuron-specific enolase (NSE), was consistently elevated in female ob/ob mice and showed a similar sex-associated pattern in female patients with non-alcoholic steatohepatitis (NASH). While the human NASH data provide correlative support rather than direct clinical validation, these observations underscore the importance of considering sex as a biological variable in metabolic disease research. Incorporating sex-specific biomarker profiles may help refine mechanistic interpretation and inform future studies toward personalized therapeutic approaches.

## 1. Introduction

Obesity constitutes a major global health burden and significantly compromises both healthy life expectancy and overall quality of life. Among the metabolic disorders closely associated with obesity, metabolic dysfunction-associated steatotic liver disease (MASLD) represents the most prevalent form of liver disease worldwide, affecting approximately 30% of the global population [1]. Notably, the age at onset has been steadily declining, with a marked increase in incidence among younger individuals presenting with obesity, diabetes, or metabolic syndrome [2]. MASLD exhibits a progressive clinical trajectory, advancing to non-alcoholic steatohepatitis (NASH), cirrhosis, and hepatocellular carcinoma in a substantial subset of patients [2]. Despite these risks, only two pharmacological treatments have been approved to date: Rezdiffra (resmetirom), approved by the FDA in March 2024, and Wegovy (semaglutide), approved in August 2025 [3,4].

Sex differences in fat distribution are well recognized, and liver diseases similarly demonstrate heterogeneity between males and females in terms of prevalence, risk, and underlying mechanisms [5]. For example, premenopausal women exhibit a lower prevalence of MASLD than men, whereas after menopause, the prevalence in women becomes comparable to or even exceeds that in men [5]. Moreover, MASLD-related heart failure risk appears to be more pronounced in women [6]. These sex-specific differences may arise not only from hormonal variation but also from molecular mechanisms, although the precise pathways remain under investigation [7,8,9,10]. Because MASLD research has been conducted predominantly using male mice, current models still have limitations in reflecting sex-dependent mechanisms of disease pathogenesis [11,12]. Therefore, incorporating sex as a biological variable and identifying sex-specific molecular biomarkers for early detection and therapeutic targeting are urgently needed in metabolic disease research.

In this study, we employed the C57BL/6J-*Lep^ob^*/*Lep^ob^* (ob/ob) mouse, which carries a spontaneous mutation in the leptin gene (ob) and exhibits hyperphagia, obesity, and diabetes-related complications. Although ob/ob mice are widely utilized in metabolic research, relatively few studies have examined sex differences in the plasma proteome. Circulating proteins serve as valuable biomarkers that reflect systemic physiological alterations, and a large human cohort study involving 1426 males and 2632 females has already reported significant sex differences in multiple biomarkers associated with metabolic disease [13]. Based on this rationale, we aimed to analyze the plasma proteome of ob/ob mice to elucidate sex-dependent molecular differences in metabolic dysfunction.

## 2. Results

**Figure 1:**
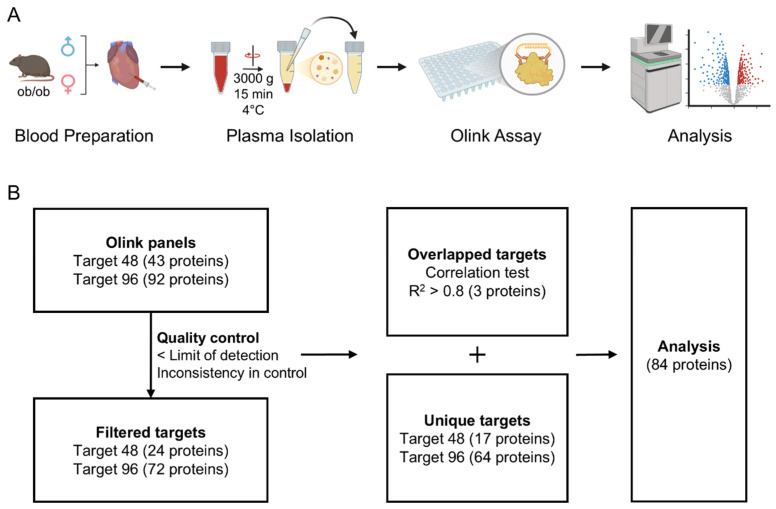
Study design and analytical pipeline for assessing sex-specific differences in ob/ob mouse plasma. (**A**) Study design. Plasma was collected from ob/ob mice and analyzed using the Olink assay, followed by downstream data processing. (**B**) Analytical pipeline. The filtering criteria that resulted in the final selection of 84 proteins are illustrated. Created with BioRender.com. Park, K. (2026) https://BioRender.com/feonp5l.

For Olink analysis, plasma samples were collected from a total of 12 ob/ob mice (6 females and 6 males) via cardiac puncture (Figure 1A). Plasma proteomic profiling was performed using the Olink Target 48 Mouse Cytokine panel and the Olink Target 96 Mouse Exploratory panel. To ensure data reliability, we applied quality control procedures to exclude proteins that were below the detection limit or showed low consistency across control samples. After removing redundant targets and retaining three proteins showing high consistency across both panels, along with 81 unique targets from the individual panels, a final dataset of 84 proteins was obtained (Figure 1B).

**Figure 2:**
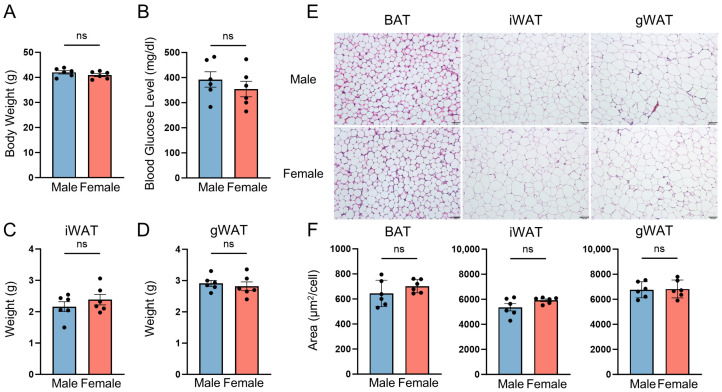
Comparable adiposity between male and female ob/ob mice. (**A**) Body weight. (**B**) Blood glucose level. (**C**) iWAT weight. (**D**) gWAT weight. (**E**) H&E images of adipose tissues. Scale bars: 50 μm for BAT and 100 μm for iWAT and gWAT. (**F**) Lipid droplet area in adipose tissues. iWAT, subcutaneous inguinal white adipose tissue; gWAT, gonadal white adipose tissue; BAT, brown adipose tissue; ns, not significant.

To minimize variability associated with body weight or adiposity, we used 8-week-old ob/ob mice whose body weights were comparable between males and females (Figure 2A). Blood glucose levels did not differ significantly between sexes (Figure 2B). Similarly, the weights of two major white adipose tissue depots showed no sex-dependent differences (Figure 2C,D). Histological examination using H&E staining revealed no sex differences (Figure 2E), and lipid droplet area in both brown and white adipose tissues was also comparable between males and females (Figure 2F). Together, these results indicate that male and female ob/ob mice exhibit similar degrees of adiposity, suggesting that subsequent proteomic differences reflect inherent biological sex differences rather than differences in body weight or adiposity.

**Figure 3:**
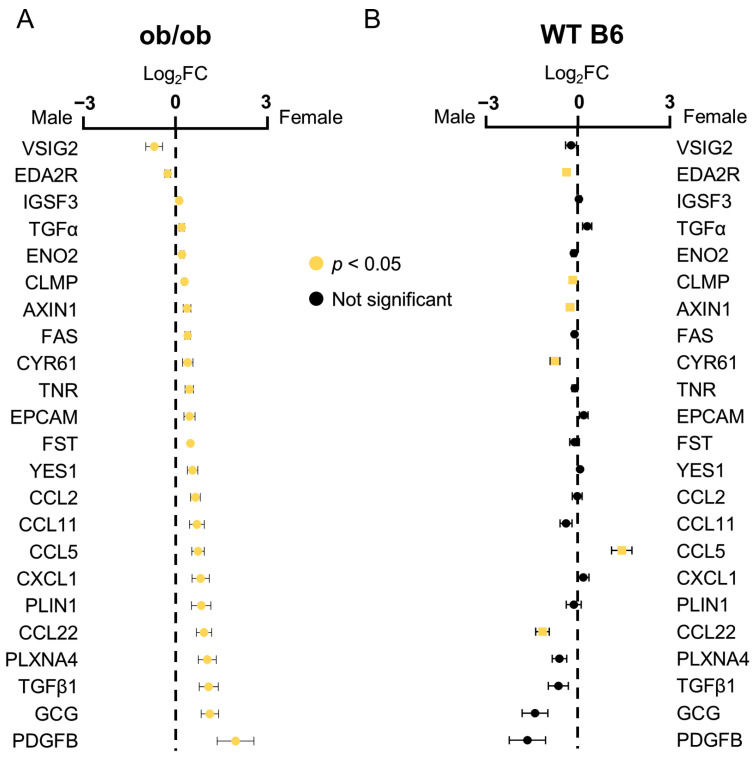
Sex-differential plasma proteins in ob/ob mice and control mice. (**A**) Comparison between male and female ob/ob mice. (**B**) Comparison between male and female wild-type C57BL/6 (WT B6) mice (Suh et al. 2025) [14]. Proteins showing statistically significant differences (*p* < 0.05) are highlighted in yellow. Fold changes (FC) were calculated from NPX values generated by the Olink assay.

Among the 84 quantified proteins, 23 exhibited sex-dependent expression patterns. Of these, two proteins (VSIG2 and EDA2R) were elevated in males, whereas 21 proteins were elevated in females (Figure 3A). Comparison with published datasets from wild-type C57BL/6 mice [14] revealed that only two proteins (EDA2R, CCL5) displayed the same sex-specific pattern (Figure 3B), suggesting that sex differences under obese, disease conditions differ substantially from those observed in normal physiological states.

**Figure 4:**
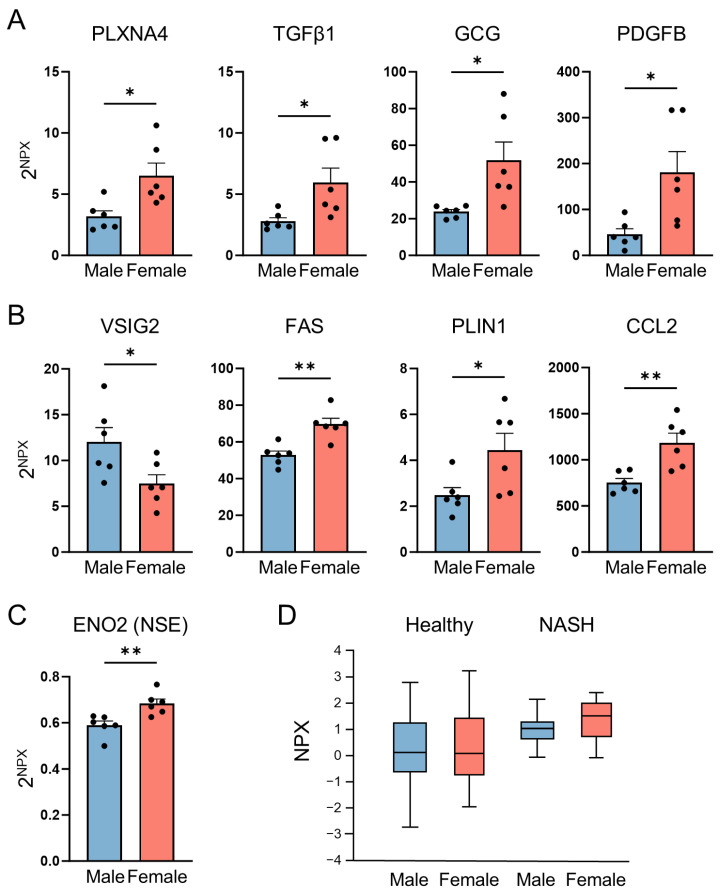
Representative plasma proteins exhibiting sex-differential expression. (**A**) Proteins showing opposite sex-dependent expression patterns between WT and ob/ob mice. (**B**) Representative proteins displaying sex differences. (**C**) Levels of ENO2 (NSE) in ob/ob mice. (**D**) Levels of ENO2 (NSE) in healthy controls and patients with NASH. Data are presented as mean ± SEM. * *p* < 0.05; ** *p* < 0.01. ENO2, Enolase 2; NSE, Neuron-specific enolase; NASH, Non-alcoholic steatohepatitis.

Notably, several plasma proteins, including PLXNA4, TGFβ1, GCG, and PDGFB, showed sex-specific expression patterns in ob/ob mice that were opposite to those previously reported in wild-type mice (Figure 4A). Additional representative proteins included VSIG2, which was elevated in males, and FAS, PLIN1, and CCL2, which were elevated in females (Figure 4B). Among these, we focused further on Enolase 2 (ENO2), also known as neuron-specific enolase (NSE). ENO2 was selected because it is already used in routine clinical practice, supporting its potential translational relevance. ENO2 is a glycolytic enzyme predominantly expressed in neurons and neuroendocrine cells and is released into the bloodstream during neuronal injury or neuroinflammation, making it a well-established biomarker [15,16]. We found that ENO2 levels were significantly elevated in female ob/ob mice (Figure 4C) and that female NASH patients also exhibited higher circulating ENO2 levels compared to healthy controls (Figure 4D).

## 3. Discussion

In this study, we analyzed sex differences in plasma protein expression using the ob/ob mouse, a well-established model of obesity. Olink-based proteomic profiling revealed that 27% (23/84) of quantified proteins exhibited sex-specific differences, with the majority (21/23) elevated in females. Notably, despite the absence of differences in body weight, blood glucose levels, or adiposity between male and female ob/ob mice, clear sex-dependent variation was observed in circulating proteins. These findings indicate that the observed differences arise from inherent biological sex variation within a body weight-controlled model.

Among the 23 proteins that displayed sex-specific regulation in obese mice, only two proteins (EDA2R and CCL5) showed consistent sex-dependent patterns in wild-type mice. This suggests that the obese pathophysiological state differs substantially from normal physiology. Indeed, proteins such as PLXNA4, TGFβ1, GCG, and PDGFB exhibited expression patterns opposite to those reported in wild-type animals, implying that interactions among hormonal signaling, inflammatory pathways, and metabolic processes may diverge between sexes under obese conditions. However, further mechanistic experiments will be required to directly elucidate the causal pathways underlying these sex-dependent differences.

For example, CCL2 (MCP-1), one of the sex-dependent proteins identified, is a chemokine closely associated with the pathogenesis of obesity and MASLD. CCL2 promotes monocyte recruitment and amplifies inflammatory responses in adipose tissue and the liver [17]. The female-specific elevation of CCL2 observed in ob/ob mice is consistent with previous findings reporting increased CCL2 levels in female mice subjected to high-fat-diet-induced obesity [18]. These observations support the idea that inflammatory responses in obesity may differ substantially between sexes.

A particularly notable finding was the significant elevation of ENO2 (NSE) in female ob/ob mice. In a study involving 901 adults, aging was associated with increased BMI, and ENO2 levels increased with age in women but decreased in men [19]. These findings suggest that sex differences in ENO2 expression become more pronounced under pathological metabolic stress rather than normal physiological conditions. Thus, ENO2 may represent a female-specific circulating biomarker with potential relevance in obesity and metabolic disease.

A limitation of this study is the relatively small sample size (n = 6 per sex) for plasma proteomic analysis. Although this number was chosen based on prior studies, the limited statistical power may affect the robustness and reproducibility of the observed sex-dependent differences. Therefore, these findings should be interpreted with caution, and future studies with larger cohorts will be necessary for independent validation.

The second limitation concerns the statistical analysis, as two-tailed Student’s t-tests were performed across 84 proteins without correction for multiple comparisons, increasing the risk of false-positive results. Therefore, the findings should be interpreted with caution, and future studies incorporating false discovery rate (FDR) adjustment and independent validation will be necessary to strengthen statistical power.

The third limitation is the absence of randomization or blinding in this study. Although it is inherently challenging in sex-based research due to the clear identification of biological sex, this absence may still introduce bias, particularly given the small cohort size. This limitation should be considered when interpreting the observed sex-dependent differences. Future studies incorporating randomization or blinding will be important to enhance experimental rigor and reduce potential sources of bias in sex-based analyses.

The fourth limitation of this study is that biomarker analyses were performed immediately after animal transport, without an acclimatization period. Acute transport-related stress may influence adipokine release and sympathetic activation, potentially affecting circulating biomarkers. Although animals were sacrificed in an alternating male–female sequence to reduce bias, this approach was insufficient to fully account for stress-related effects. Accordingly, future studies incorporating acclimatization periods will be important to improve experimental rigor.

The fifth limitation is that although the comparison between ob/ob mice and previously published wild-type datasets is conceptually informative, differences in experimental conditions, age, and analytical pipelines may confound direct biological interpretation. Therefore, these cross-study comparisons should be considered exploratory and interpreted with caution. Future studies with matched experimental designs will be required for more robust biological comparisons.

The sixth limitation is that sex-dependent differences were observed at the plasma proteomic level; however, no substantial differences in body weight, blood glucose levels, or adiposity were detected between male and female ob/ob mice in this study. This may be partly due to the relatively young age of the animals (8 weeks), as phenotypic or organ-level functional differences may become apparent with longer disease duration. Accordingly, future studies incorporating detailed phenotypic and organ function assessments will be needed to clarify the pathophysiological significance of the observed sex-specific biomarker patterns.

Our plasma proteomic analysis in the ob/ob obesity model revealed sex-dependent differences in circulating protein expression profiles. These findings underscore the importance of incorporating sex as a biological variable in metabolic disease research. While certain proteins, including ENO2, have been identified, their potential utility as diagnostic or prognostic biomarkers remains to be determined. Further mechanistic studies and independent validation will be required to clarify their biological relevance and to assess their applicability in future translational research.

## 4. Materials and Methods

### 4.1. Animals

ob/ob mice (JAX #000632, B6.Cg-*Lep^ob^*/J) were obtained from The Jackson Laboratory via Orient Bio Inc. (Seongnam City, Republic of Korea). The study utilized two groups of 8-week-old animals (total n = 12): male ob/ob mice (n = 6) and female ob/ob mice (n = 6).

The sample size was determined with reference to previous studies and set at the minimum number required to obtain meaningful results. No criteria for including and excluding animals were set, and there were no exclusions. No randomization was used. Blinding was not performed because sex could be readily identified by visual examination. Experiments were performed immediately after transport; therefore, no additional housing was required. There were no expected or unexpected adverse events or humane endpoints. To minimize potential confounders, we performed sampling in an alternating male–female sequence.

All animals were in a fed state at the time of blood collection. Euthanasia was performed via CO_2_ exposure without the use of additional anesthesia. Blood was obtained through cardiac puncture and collected into EDTA-coated microtubes (BD, Cat# 365974, Franklin Lakes, NJ, USA). Following gentle inversion, the samples were centrifuged at 3000× *g* for 15 min at 4 °C. The resulting plasma supernatant was carefully collected and stored at −80 °C until further analysis.

Body weight and adipose tissue weights were measured to assess the degree of adiposity in mice. Blood glucose was determined prior to euthanasia using blood obtained by tail snip, and each measurement was performed twice with a glucometer; the mean value was used for analysis. Subcutaneous inguinal white adipose tissue (iWAT), gonadal white adipose tissue (gWAT), and brown adipose tissue (BAT) were excised, and the weights of the combined bilateral tissues were recorded. Tissue sectioning (5 μm thickness) and hematoxylin and eosin (H&E) staining were outsourced to the Morphology Core Facility at Yonsei University Wonju College of Medicine. Lipid droplet areas in each tissue were quantified using ImageJ software (version 1.54 g).

The animal study protocol was approved by the Institutional Animal Care and Use Committee (IACUC) of Yonsei University Wonju College of Medicine (Approval Code: YWC-250519-1 and Approval Date: 29 May 2025).

### 4.2. Olink Analysis

Plasma protein concentrations were measured using the Olink Target 48 Mouse Cytokine panel (#93400) and the Olink Target 96 Mouse Exploratory panel (#95380). Both panels were processed and analyzed by Macrogen Inc. (Seoul City, Republic of Korea). Protein abundances were reported as NPX (Normalized Protein Expression) values generated by the Olink platform, expressed on a log_2_ scale. For improved interpretability and to facilitate comparison with the conventional qRT-PCR ΔΔCt method, NPX values were transformed to 2^NPX^ for downstream analyses.

### 4.3. Human Protein Atlas Dataset

Publicly available Olink plasma proteomics data were used, originating from plasma samples collected from 2457 patients enrolled in 32 cohorts. These data are integrated into the Pan-disease Extended Blood Protein Profiles (Olink HT) resource within the Blood section of the Human Protein Atlas.

### 4.4. Statistics

Statistical analysis was conducted using a two-tailed Student’s *t*-test in Microsoft Excel, and *p*-values (*p*) below 0.05 were considered statistically significant. All data are expressed as mean ± SEM.

## Data Availability

The datasets presented in this article are not readily available because the data are part of an ongoing study.
